# Identification of *bakanae* disease resistance loci in *japonica* rice through genome wide association study

**DOI:** 10.1186/s12284-017-0168-z

**Published:** 2017-06-08

**Authors:** Andrea Volante, Alessandro Tondelli, Maria Aragona, Maria Teresa Valente, Chiara Biselli, Francesca Desiderio, Paolo Bagnaresi, Slavica Matic, Maria Lodovica Gullino, Alessandro Infantino, Davide Spadaro, Giampiero Valè

**Affiliations:** 1 Council for Agricultural Research and Economics (CREA), Rice Research Unit, S.S. 11 to Torino, Km 2.5, 13100 Vercelli, Italy; 2 Council for Agricultural Research and Economics (CREA), Genomics Research Centre, Via S. Protaso, 302, 29017 Piacenza, Fiorenzuola d’Arda Italy; 3 Council for Agricultural Research and Economics (CREA), Plant Pathology Research Centre, Via C. G. Bertero 22, 00156 Roma, Italy; 4 AGROINNOVA, Università di Torino, Largo P. Braccini 2, 10095 Torino, Grugliasco Italy; 5 DISAFA, Università di Torino, Largo P. Braccini 2, 10095 Torino, Grugliasco Italy

**Keywords:** *Bakanae*, Disease resistance loci, Genome wide association study (GWAS), Candidate genes

## Abstract

**Background:**

*Bakanae* disease, caused by seed-borne *Fusarium* species, mainly *F. fujikuroi*, is a rice disease whose importance is considerably increasing in several rice growing countries, leading to incremental production losses.

**Results:**

A germplasm collection of *japonica* rice was screened for *F. fujikuroi* resistance, allowing the identification of accessions with high-to-moderate levels of resistance to *bakanae*. A GWAS approach uncovered two genomic regions highly associated with the observed phenotypic variation for response to *bakanae* infection on the short arm of chromosome 1 (named as *qBK1_628091*) and on the long arm of chromosome 4 (named as *qBK4_31750955*). High levels of phenotypic resistance to *bakanae* were associated to the cumulated presence of the resistant alleles at the two resistance loci, suggesting that they can provide useful levels of disease protection in resistance breeding. A fine comparison with the genomic positions of qBK1_628091 and qBK4_31750955 with respect to the QTLs for *bakanae* resistance reported in the literature suggests that the resistant loci here described represent new genomic regions associated to *F. fujikuroi* resistance. A search for candidate genes with a putative role in *bakanae* resistance was conducted considering all the annotated genes and *F. fujikuroi*-related DEGs included in the two genomic regions highlighting several gene functions that could be involved in resistance, thus paving the way to the functional characterization of the resistance loci.

**Conclusions:**

New effective sources for *bakanae* resistance were identified on rice chromosomes 1 and 4 and tools for resistance breeding are provided.

**Electronic supplementary material:**

The online version of this article (doi:10.1186/s12284-017-0168-z) contains supplementary material, which is available to authorized users.

## Background

*Bakanae* disease is one of the most serious and oldest problems affecting rice production, first described in 1828 in Japan (Ito and Kimura 1931) and currently identified in Europe, Asia, Africa, and North America (Ou 1985; Pra et al. 2010). In various rice growing countries, significant yield losses caused by the disease can range from 50% to more than 70% (Ou 1985; Rood 2004). Increasing *bakanae* disease incidence has been reported in Italy (Amatulli et al. 2012) and major growing areas of Asia such as Pakistan, South Korea, Bangladesh, Northern India, and Taiwan (Khan et al. 2000; Park et al. 2009; Haq et al. 2011; Gupta et al. 2014; Chen et al. 2016).

*Bakanae* is caused by one or more seed-borne *Fusarium* species, mainly *F. fujikuroi* (Wulff et al. 2010), and the disease may infect rice plants from the pre-emergence stage to the mature stage, with severe infection of rice seeds resulting in poor germination or withering (Iqbal et al. 2011). *F. fujikuroi* belongs to hemibiotrophs *fungi*, whose initial infection relies on a living host (biotrophic), and progressive infection involves a consumption and destruction of the host cells (necrotroph; Ma et al. 2013). Seeds contaminated with the *fungus* provide initial *foci* for primary infection. Under favorable environmental conditions, infected plants have the capacity to produce numerous *conidia* that subsequently infect proximate healthy panicles through aerial conidial diffusion by wind, producing infected seeds (Ou 1985; Ora et al. 2011; Matic et al. 2017). During primary infection, mature rice plants are tall, frequently stunted, with an angle of leaf insertion wider than in healthy seedlings. Moreover, infected plants eventually die, while panicles on surviving plants do not develop any grains, thus resulting in yield loss (Desjardins et al. 2000; Mew and Gonzales 2002; Ou 1985).

The altered plant morphology is due to the ability of *F. fujikuroi* to produce and secrete gibberellic acids (GAs) (Bearder 1983; Ou 1985). Although GAs are considered as secondary metabolites (SMs) in *Fusarium* because they are not essential for fungal growth and development, they are thought to contribute to the virulence of *F. fujikuroi*, the only *Fusarium* species capable of GAs biosynthesis, by controlling jasmonic acid-responsive gene expression and jasmonic acid-mediated plant immune responses (Wiemann et al. 2013; Siciliano et al. 2015). GA production was also associated with fungicide sensitivity of different *F. fujikuroi* isolates (Tateishi et al. 1998; Tateishi and Suga 2015).

The most common management practices to limit *bakanae* are based on thermal seed treatment (hot water immersion) or fungicides. The hot water immersion method (Hayasaka et al. 2001) was demonstrated ineffective on severely infected rice seeds, because thermal effect is not efficiently transmitted to the pericarp layers. Also seed dressing with fungicides has restricted efficiency in destroying the spores of the *fungus*, owing their resistance to several fungicides (Iqbal et al. 2011; Park et al. 2009; Kim et al. 2010; Lee et al. 2011). Promising results have only recently been obtained through a combination of antagonistic yeasts and thermotherapy (Matic et al. 2014). However, the current incidence of *bakanae* disease is increasing, leading to serious concerns in the main rice-producing areas worldwide (Wahid et al. 1993; Ma et al. 2008) and there is a strong request for alternative disease control measures, such as the identification of rice *bakanae* resistant cultivars (cvs.). However, only a few accessions were reported to effective source of resistance to *bakanae*. An extensive search carried out on more than 400 rice accessions identified only one and 12 cvs. with high and moderate resistance, respectively (Li et al. 1993). Similarly, in other studies only a few resistant varieties were identified after application of different screening procedures (Lv 1994; Khokhar and Jaffrey 2002; Kim et al. 2014).

Knowledge on mapped loci conferring resistance to *bakanae* is very limited. Two QTLs for *bakanae* resistance derived from the Chinese *japonica* cv. Chunjiang 06 were identified on chromosomes 1 and 10, explaining each one about 13% of phenotypic variation (Yang et al. 2006). Hur et al. (2015), using near-isogenic lines (NILs) derived from a cross between the highly resistant *indica* variety Shingwang and the *japonica* susceptible variety Ilpum, identified a major QTL, named as *qBK1*, on the long arm of chromosome 1 explaining 65% of the phenotypic variation and not coincident with the QTL identified in Chunjiang 06. More recently, three QTLs were identified on chromosome 1 (Fiyaz et al. 2016). Two of them (*qBK1.2* and *qBK1.3*), detected on the short arm of chromosome 1, represent novel QTLs, while the third one (*qBK1.1*) was mapped in coincidence with the *qBK1* QTL described by Hur et al. (2015).

A large genetic diversity of the pathogen population has been highlighted for strains isolated from Asia, Africa and Europe (Wulff et al. 2010; Jeon et al. 2013; Valente et al. 2016), therefore supporting the necessity of additional loci conferring rice resistance to *bakanae*. Whole-genome association mapping (GWAS) has recently demonstrated to offer better resolution than QTL mapping, thus reducing the QTL interval of confidence and, consequently, the number of candidate genes underlying individual QTLs (Huang et al. 2010, 2011; Courtois et al. 2013). Linkage disequilibrium (LD) decay, which determines the expected resolution in the GWAS approach, has been reported to range from 500 kb in the temperate *japonica* rice background to 75 kb in the *indica* background (Huang et al. 2010; Mather et al. 2007), even considering that in germplasm collection of more related temperate *japonica* rice accessions values of LD decay of 1250 kb were also observed (Biscarini et al. 2016).

The main objective of the present study was the screening of a *japonica* rice germplasm collection for *bakanae* resistance after artificial inoculation with a virulent isolate of *F. fujikuroi*, in order to map the genetic polymorphisms underlying rice resistance against this disease. Genome-wide association study allowed the identification and localization of two new QTLs conferring rice resistance to *bakanae* disease in the rice *japonica* background.

## Results

### *Bakanae* disease resistance in the Rice Germplasm Collection

**Fig. 1:**
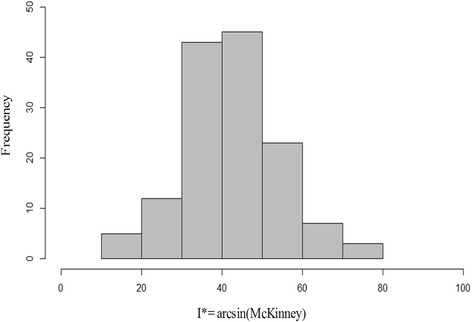
Frequency distribution of *bakanae* disease resistance (I*) in the Rice Germplasm Collection analyzed in the present study

A virulent *F. fujikuroi* isolate was used to inoculate seeds of 138 rice accessions adapted to the Italian growing conditions, in order to explore their resistance to *bakanae* disease. A weak skewing of the frequency distribution towards high levels of susceptibility was observed in the collection (Fig. 1); nevertheless, some accessions showed high-to-moderate resistance (e.g. the tropical *japonica* genotypes: Greppi, L205, Arsenal, IAC32_52 and Adair with a disease index (I*) value <20, Table 1). Highly significant genotypic variation for the trait under study was detected by ANOVA (Table 2), and confirmed by the calculated H^2^ value of 0.84. Temperate *japonica* accessions showed a higher incidence of the disease with respect to tropical *japonica* accessions (average I* values 42.8 and 40.1, respectively). However the difference was found to be not statistically significant (data not shown).

**Table 1:** List of accessions in the Rice Germplasm Collection assembled for this work

Accession Name	Sub-population	Origin	I*	S1_628091	S4_31750955	Structure group
GREPPI	Tropical Japonica	Italy	13.2	A	C	adm
L205	Tropical Japonica	USA	16.5	A	C	2
ARSENAL	Tropical Japonica	Italy	17.9	A	C	adm
IAC32 52	Tropical Japonica	Brazil	18.4	A	C	2
ADAIR	Tropical Japonica	USA	19.1	A	C	2
KING	Tropical Japonica	Italy	22.8	A	C	2
MAIORAL	Temperate Japonica	Portugal	23.0	A	C	adm
GRALDO	Tropical Japonica	Italy	24.0	T	C	adm
BENGAL	Temperate Japonica	USA	25.2	A	C	adm
ALAN	Tropical Japonica	USA	25.7	A	C	2
MAYBELLE	Tropical Japonica	USA	26.0	A	C	2
A201	Tropical Japonica	USA	26.8	A	C	2
ITALPATNA x MILYANG	Temperate Japonica	Portugal	28.9	T	C	adm
FLIPPER	Temperate Japonica	Italy	29.5	T	C	1
DELLROSE	Tropical Japonica	USA	29.7	A	C	2
ERCOLE	Temperate Japonica	Italy	29.9	T	C	1
AUGUSTO	Temperate Japonica	Italy	30.0	T	C	1
DUCATO	Temperate Japonica	Italy	30.1	T	C	1
LUXOR	Temperate Japonica	Italy	30.1	T	C	1
CARRICO	Temperate Japonica	Portugal	30.6	T	C	1
HARRA	Temperate Japonica	Australia	30.6	T	C	1
LAGRUE	Tropical Japonica	USA	31.2	A	-	2
LORD	Temperate Japonica	Italy	31.4	T	C	1
LIDO	Temperate Japonica	Italy	31.4	T	C	1
HAREM	Temperate Japonica	Portugal	31.6	T	C	1
CASTELMOCHI	Temperate Japonica	Italy	32.6	T	C	1
GZ8367	Temperate Japonica	Egypt	32.7	T	C	1
JUBILIENI	Temperate Japonica	Bulgary	32.8	T	C	1
DELFINO	Temperate Japonica	Italy	34.1	T	C	1
BRAZOS	Tropical Japonica	USA	34.3	T	-	2
VENERE	Temperate Japonica	Italy	34.4	T	C	1
CT36	Temperate Japonica	Colombia	34.6	T	C	1
FRANCES	Temperate Japonica	Spain	34.9	T	C	1
GRITNA	Temperate Japonica	Italy	35.2	T	C	1
CT58	Temperate Japonica	Colombia	35.2	T	C	1
LUNA	Temperate Japonica	USA	35.4	T	C	1
CRESO	Temperate Japonica	Italy	35.5	A	C	1
BARAGGIA	Temperate Japonica	Italy	35.6	T	G	1
COLINA	Temperate Japonica	Spain	35.7	T	C	1
ITALMOCHI	Temperate Japonica	Italy	35.7	T	C	1
GRAAL	Tropical Japonica	France	35.9	T	C	adm
PECOS	Tropical Japonica	USA	36.0	T	G	adm
CIGALON	Temperate Japonica	France	36.2	T	C	1
SALVO	Tropical Japonica	Italy	36.5	T	C	adm
BIANCA	Temperate Japonica	Italy	36.7	T	C	1
LUSITO IRRADIADO	Temperate Japonica	Portugal	36.8	T	C	1
KULON	Temperate Japonica	Russia	36.8	T	C	1
GIGANTE VERCELLI	Temperate Japonica	Italy	36.9	T	G	1
LADY WRIGHT	Tropical Japonica	USA	37.1	T	G	adm
MANTOVA	Temperate Japonica	Italy	37.6	T	C	1
BAHIA	Temperate Japonica	Spain	38.4	T	C	1
ALICE	Temperate Japonica	Italy	38.5	T	C	1
CENTAURO	Temperate Japonica	Italy	38.7	T	C	1
EUROPA	Temperate Japonica	Italy	38.8	T	C	1
ESTRELA	Temperate Japonica	Portugal	38.9	T	C	adm
IBO 400	Temperate Japonica	Portugal	38.9	A	C	1
DIMITRA	Temperate Japonica	Greece	38.9	T	C	1
CLOT	Temperate Japonica	Spain	39.0	T	C	1
L201	Tropical Japonica	USA	39.1	A	-	2
JEFFERSON	Tropical Japonica	USA	39.3	A	G	2
L202	Tropical Japonica	USA	40.0	A	C	2
EUROSE	Temperate Japonica	Italy	40.3	T	C	1
L204	Tropical Japonica	USA	40.5	A	C	2
BALILLA	Temperate Japonica	Italy	40.5	T	C	1
CHIPKA	Temperate Japonica	Bulgary	40.6	T	C	1
LUCERO	Temperate Japonica	Italy	40.7	T	C	1
GIANO	Tropical Japonica	Italy	41.0	T	C	adm
DRAGO	Temperate Japonica	Italy	41.0	T	C	1
CINIA 40	Temperate Japonica	Cile	41.1	T	C	1
SAKHA 103	Temperate Japonica	Egypt	41.1	T	C	1
GARDE SADRI	Temperate Japonica	Turkey	41.7	T	C	1
BELLE PATNA	Tropical Japonica	USA	41.7	A	C	2
LAMONE	Tropical Japonica	Italy	41.8	T	C	2
FAMILIA 181	Temperate Japonica	Portugal	42.0	T	C	1
CORBETTA	Temperate Japonica	Italy	42.1	T	C	1
EUROSIS	Temperate Japonica	Italy	42.3	A	C	adm
ALPE	Temperate Japonica	Italy	42.3	T	C	1
CARIOCA	Tropical Japonica	Italy	42.4	T	C	adm
VIALONE NANO	Temperate Japonica	Italy	42.5	T	G	1
LOMELLINO	Temperate Japonica	Italy	42.7	T	C	1
KORAL	Temperate Japonica	Italy	42.7	T	C	1
SAKHA 102	Temperate Japonica	Egypt	43.3	T	C	1
ERMES	Tropical Japonica	Italy	43.4	T	G	adm
BURMA	Tropical Japonica	Italy	43.4	T	C	adm
CALMOCHI 101	Temperate Japonica	USA	43.5	T	C	1
ANTONI	Temperate Japonica	Bulgary	44.1	T	C	1
GUADIAMAR	Temperate Japonica	Spain	44.2	T	C	1
ARGO	Temperate Japonica	Italy	44.4	T	C	1
CARNISE	Temperate Japonica	Italy	44.5	T	G	1
AMERICANO 1600	Temperate Japonica	Italy	44.9	T	C	1
ARBORIO	Temperate Japonica	Italy	45.1	T	G	1
DIXIEBELLE	Tropical Japonica	USA	45.3	T	C	2
M203	Temperate Japonica	USA	45.6	T	C	1
BALDO	Temperate Japonica	Italy	45.6	T	C	1
ARTEMIDE	Tropical Japonica	Italy	46.0	T	C	adm
BOMBON	Temperate Japonica	Spain	46.1	T	C	1
CARINA	Temperate Japonica	Bulgary	46.4	T	G	1
GANGE	Tropical Japonica	Italy	46.5	T	C	2
ALEXANDROS	Tropical Japonica	Greece	46.7	A	C	2
LACASSINE	Tropical Japonica	USA	46.7	T	C	2
M6	Temperate Japonica	Italy	48.3	T	G	1
MARATELLI	Temperate Japonica	Italy	48.3	T	C	1
FORTUNA	Tropical Japonica	Italy	49.0	T	G	adm
AIACE	Tropical Japonica	Italy	49.5	T	C	2
BAIXET	Temperate Japonica	Spain	49.8	T	C	1
GIZA 177	Temperate Japonica	Egypt	50.3	T	C	1
CRIPTO	Temperate Japonica	Italy	50.7	T	C	1
AGOSTANO	Temperate Japonica	Italy	51.0	T	C	1
CAPATAZ	Temperate Japonica	Spain	51.3	T	C	1
ANSEATICO	Temperate Japonica	Italy	51.3	T	C	adm
BONNI	Temperate Japonica	Italy	51.4	T	C	1
BALZARETTI	Temperate Japonica	Italy	52.2	T	C	1
BERTONE	Temperate Japonica	Italy	52.2	T	C	1
TEXMONT	Tropical Japonica	USA	52.6	T	-	2
KARNAK	Temperate Japonica	Italy	54.3	T	G	1
CARNAROLI	Temperate Japonica	Italy	54.3	T	G	1
AKITAKOMACHI	Temperate Japonica	Japan	54.5	T	C	1
IBO 380–33	Temperate Japonica	Portugal	54.5	T	G	1
BOMBILLA	Temperate Japonica	Spain	54.5	T	-	1
ALLORIO	Temperate Japonica	Italy	54.6	T	C	1
DREW	Tropical Japonica	USA	54.8	T	C	2
M202	Temperate Japonica	USA	55.1	T	C	1
HANDAO 297	Temperate Japonica	China	55.3	T	G	1
ITALPATNA 48	Temperate Japonica	Italy	55.4	T	C	1
GIOVANNI MARCHETTI	Temperate Japonica	Italy	55.7	T	G	1
ARIETE	Temperate Japonica	Italy	56.0	T	C	1
COCODRIE	Tropical Japonica	USA	57.0	T	C	2
HONDURAS	Tropical Japonica	Spain	59.9	T	G	2
CAMPINO	Temperate Japonica	Portugal	60.1	T	C	1
CARMEN	Temperate Japonica	Italy	60.8	T	C	1
ALPHA	Temperate Japonica	Italy	61.2	T	G	1
CALENDAL	Temperate Japonica	France	61.6	T	C	1
M204	Temperate Japonica	USA	62.9	T	C	1
SELENIO	Temperate Japonica	Italy	66.6	T	C	1
A301	Tropical Japonica	USA	68.7	T	C	2
GLADIO	Tropical Japonica	Italy	73.4	T	C	2
ESCARLATE	Temperate Japonica	Portugal	77.0	T	G	1
JACINTO	Tropical Japonica	USA	77.7	T	C	2

Values of the disease scoring (I*) and the allelic status for the peak SNP markers (S1_628091 and S4_31750955) of the two identified *bakanae* resistance QTL is reported. Structure groups = groups defined by Structure (membership ≥70%)

**Table 2:** Analysis of variance for the *bakanae* disease resistance test carried-out on the Rice Germplasm Collection

Source	DF	SS	MS	F - value	P(F)
Replicate	2	65.35	32.67	1.31	0.272
Genotype	137	52,013.95	379.66	15.2	<0.001
Residual	240	5992.82	24.97		
Total	379	58,072.12	153.22		

### Population structure of the Rice Germplasm Collection and genetic diversity analysis

The model based analysis of the panel structure was performed with Structure coupled with Structure Harvester analysis of the results, taking into account also the number of admixed varieties identified at each K value, as proposed by Courtois et al. (2012). Both the plot of Ln(K) and the analysis of ΔK against increasing K values indicated K = 2 as the most probable value (Additional file 1: Figure S1). At K = 2, the percentage of varieties classified as admixed was 13.7%, while for higher K values the percentage raised to over 43% (data not shown). The Structure analysis at K = 2 identified a subpopulation (91 accessions) constituted by varieties derived from the temperate *japonica* subspecies, and a second group (28 accessions) including tropical *japonica*-derived varieties (Table 1).

**Fig. 2:**
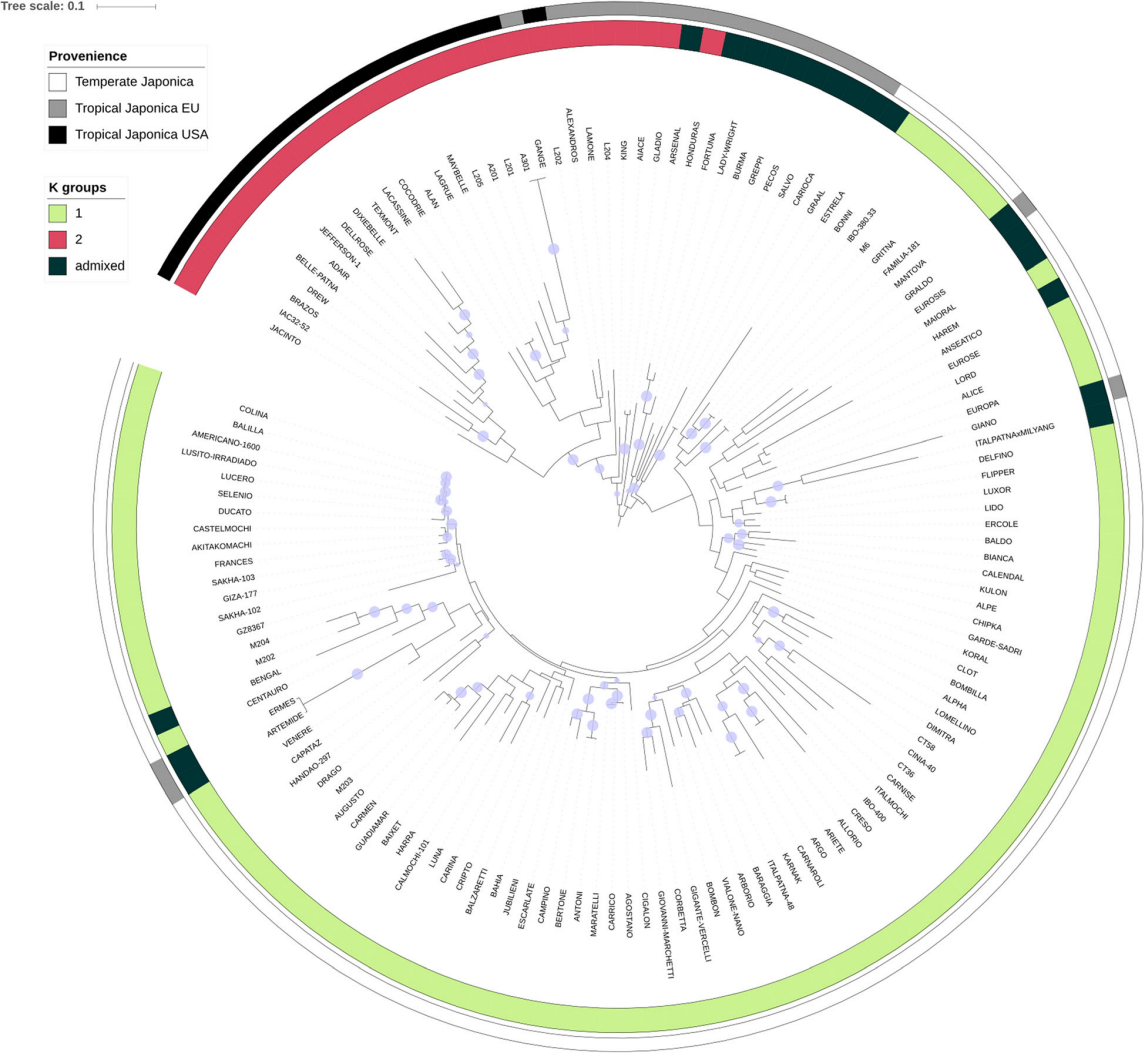
Neighbor-joining tree of the Rice Germplasm Collection. On each branch the *blue circles* show the results of the bootstrap analysis, when higher than 0.7. The outer *white-to-black coded cycle* represents the clustering of the different varieties of the panel according to *O. sativa* classification; the *inner cycle* (three-color scaled) reports the cluster organization resulting from the STRUCTURE analysis

The information obtained from Structure and integrated with the neighbor - joining tree, together with the information available in the literature are presented in Fig. 2. The three sources of information are in good accordance, with few exceptions. Artemide, Ermes, Giano and Graldo were grouped in the temperate *japonica* cluster, although these varieties were classified in the literature as tropical *japonica*. In the Structure analysis these varieties were considered as admixed. A significant proportion of European varieties documented as tropical-derived were classified as admixed in the Structure analysis, probably reflecting the contribution of cvs. from different groups in their breeding programs. The Structure output and the information taken from the literature were also crossed with results from a Principal Coordinate Analysis (PCoA) (Additional file 2: Figure S2). The first and second coordinates accounted together for 42% of the total variability (35.9 and 5.9% respectively). Principal coordinate 1 separated the sub-populations defined by the Structure analysis at K = 2 and corresponding to the temperate and tropical *japonica* groups, with the admixed accessions clustering in between. No further clustering was evident with this analysis. According to the results of this whole dataset, the LD analysis was performed assuming the panel to be structured in two subgroups.

Analysis of the genetic diversity indicated that the rice panel as a whole explained a genetic diversity of H = 0.31 while among temperate and tropical *japonica* (H = 0.23 and H = 0.27 respectively) as well as between the groups obtained by Structure analysis (0.22 and 0.23 respectively) the values were comparable (Additional file 3: Table S1 A). Within the tropical accessions, the European genotypes explained a higher diversity compared to USA accessions (H = 0.32 and H = 0.22 respectively). The genetic divergence between the temperate and tropical *japonica* of the rice panel estimated as FST (Additional file 3: Table S1 B), identified a value equal to 0.38. Considering the two tropical *japonica* subgroups (European and USA), the higher divergence was detected between the temperate *japonica* and tropical *japonica* USA (FST = 0.47). Similar divergence estimates were computed considering the two groups identified by the Structure analysis, since the FST value was equal to 0.49. All comparisons performed were significant at *p* = 0.01.

### Analysis of linkage disequilibrium and association mapping of *bakanae* resistance loci

The analysis of LD decay for each chromosome evidenced an average value of 1992 Kb (ranging from 1015 Kb for chromosome 6 to 2725 Kb for chromosome 12, Table 3). The set of markers available for GWAS after filtering by call rate and minor allele frequency, consisted of 31,752 SNPs, with a number of SNP markers per chromosome ranging from 1585 (chromosome 9) to 3970 (chromosome 1) (Table 3). Considering a total estimated genome size of 373 Mbp, we calculated a marker density of 0.09 SNP/Kbp in the whole population, with this value decreasing to 0.05, 0.06 and 0.04 in the temperate *japonica*, tropical *japonica* from Europe and tropical *japonica* from USA, respectively (Additional file 3: Table S1 C). Taking into account the extent of LD decay observed, this panel can be considered suitable to find markers associated to the resistance/susceptibility phenotype.

**Table 3:** For each chromosome the number of markers used for GWAS analysis and the corresponding average LD is indicated

Chromosome #	Number of SNPs	Average LD (Kb)
1	3970	1155
2	1997	2005
3	2389	2185
4	2746	2575
5	2438	2555
6	2505	1015
7	2703	1635
8	2530	2105
9	1585	1785
10	3169	1745
11	2696	2425
12	3024	2725
Total	31,752	

**Fig. 3:**
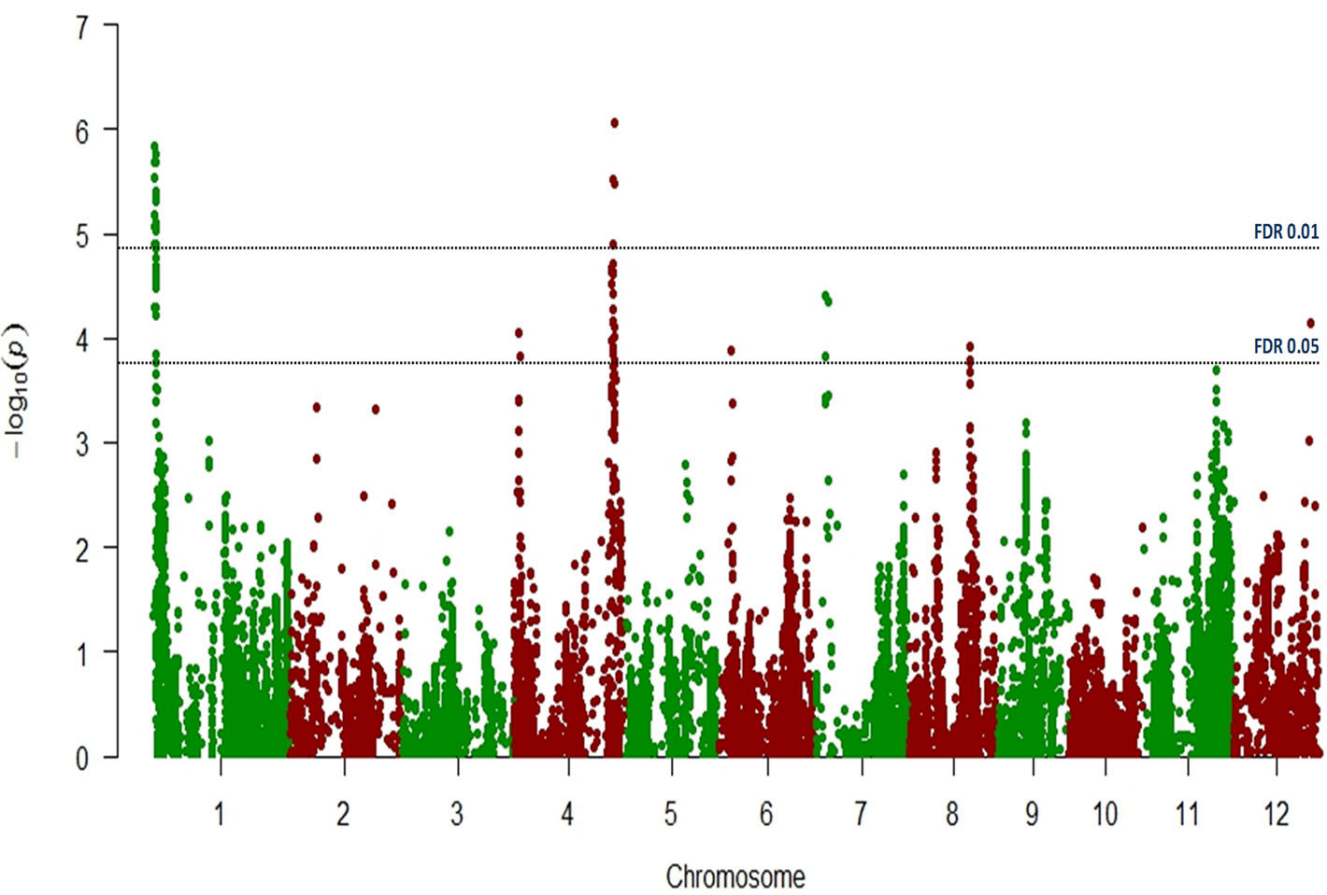
Manhattan plot showing the results of the Genome-Wide Association scan for *bakanae* disease resistance in the Rice Germplasm Collection. The −log_10_(p) from the GWA scan is plotted against the physical SNP positions on the 12 rice chromosomes. Two different FDR thresholds are indicated by *dashed horizontal lines*

Genome-wide association analysis revealed two genomic regions highly associated with the observed phenotypic variation on the short arm of chromosome 1 (*qBK1_628091*) and the long arm of chromosome 4 (*qBK4_31750955*), respectively (Fig. 3). On chromosome 1, 56 SNPs encompassing 413 Kb passed a stringent FDR threshold of 0.01 [−log_10_(*p*-value) = 4.87] (Table 4). The most associated marker mapped at the distal border of this region, at position 628,091; more distally, the observed marker coverage was low, with only two additional SNPs detected in the panel under study, at positions 170,244 and 330,484 (Additional file 4: Figure S3); none of them was associated to *bakanae* resistance. Twenty-two accessions out of 138 (15.9%) carried the resistance “A” allele at position 628,091 and they showed an average I* value of 30. This allele was more abundant in tropical *japonica* accessions (17 out of 41, 41.5%) than in temperate *japonica* ones (5 out of 97, 5.1%). Lines carrying the alternative “T” allele had an average I* score of 44.4 (Table 4; Additional file 5: Figure S4).

**Table 4:** Summary of significant marker-trait associations identified for *bakanae* disease resistance

Marker	Chr	Position (bp)	MAF	-log_10_(p)	Average I* value	
					Minor allele^a^	Major allele^b^
**S1_628091**	1	628,091	0.16	5.84	30.0	44.4
**S4_31750955**	4	31,750,955	0.15	6.06	48.8	41.1
S4_1212877	4	1,212,877	0.13	4.06	50.9	40.6
S6_4318697	6	4,318,697	0.37	3.89	43.1	41.5
S7_3180670	7	3,180,670	0.36	4.41	44.7	40.6
S8_19138386	8	19,138,386	0.24	3.92	46.6	40.6
S12_24321230	12	24,321,230	0.15	4.14	33.1	43.7

SNP markers passing the 0.01 FDR threshold are in bold

^a^Average I* value of the accessions carrying the allele at lower frequency

^b^Average I* value of the accessions carrying the allele at higher frequency

On chromosome 4, a genomic region of 595 Kb (from position 31,162,467 to position 31,757,436) was delimited by four significant SNPs (Additional file 4: Figure S3). The peak marker [−log_10_(*p*-value) = 6.06] mapped at 31,750,955, with the “C” allele associated to a lower incidence of the disease (average I* = 41.1) and harbored by 112 accessions (i.e. 85% of the whole population, 82% of the temperate *japonica* and 83% of the tropical *japonica*). The average I* value associated to the alternative “G” allele was 48.8 (Table 4; Additional file 5: Figure S4).

Eleven out of the 12 most resistant genotypes with I* value <27 carried the “A”-“C” combination at the two major loci detected. With the exception of the temperate *japonica* cvs. Bengal and Maioral, the other 10 genotypes belonged to the tropical *japonica* sub-population and were originating from the United States (5 accessions), Italy (4 accessions) and Brazil (1 accession). Sequences surrounding the SNPs associated to *bakanae* resistance on chromosomes 1 and 4 are provided in Additional file 6: Figure S5.

Additional SNPs on chromosomes 4, 6, 7, 8 and 12 were significantly associated to *bakanae* resistance at a FDR threshold <0.05 (with −log_10_(*p*-value) ranging from 3.89 to 4,41; Table 4). They may represent further loci responsible for partial levels of resistance and contributing to the observed quantitative variation for *F. fujikuroj* resistance.

### Identification of candidate genes for the major *bakanae* resistance loci

SNP markers with −log_10_(*p*-value) above the considered FDR threshold delimited the two major *bakanae* resistance loci to about 413 kbp on chromosome 1 (from position 628,091 to 1,040,823) and about 595 kbp on chromosome 4 (from position 31,162,467 to 31,757,436) (Additional file 4: Figure S3). A search for candidate genes with a putative role in *bakanae* resistance was carried out considering all the annotated genes included in the above indicated genomic regions through the screening of the *O. sativa* genomic reference sequence (Os-Nipponbare-Reference-IRGSP-1.0).

For *qBK1_628091*, 129 genes were identified in the genomic region surveyed for candidates, of which 45 were functionally annotated (Additional file 7: Table S2). No candidate genes were identified in the reference genome for the position of the most associated SNP marker (position 628,091 bp), as well as in the interval from 628,091 to 645,598 bp, this last position corresponding to the region in which the first candidate was identified; indeed in this interval only 5 genes with unknown function are annotated on the Nipponbare reference genome. Among the genes with known function, 33 *loci* encoded for protein kinases and, in particular, 22 for receptor-like kinases, of which 3 represented the receptor kinase LRK14 (Os01g0114900, Os01g0115600 and Os01g0117700) and other 3 the receptor kinase LRK10 (Os01g0117100, Os01g0117300 and Os01g0117500). These last receptors are assigned to the wheat leaf rust kinase (WLRK) receptor family, implicated in leaf rust resistance response in wheat (Feuillet et al. 1998). In addition, 2 genes (Os01g0112800 and Os01g0113150) corresponded to disease resistance proteins domain containing protein (Additional file 7: Table S2).

A total of 56 functionally annotated genes were identified in the genomic interval analyzed for *qBK4_31750955*. Also in this genomic region, several genes with functions compatible with disease resistance were identified (Additional file 7: Table S2). These included Os04g0620800, encoding for a NB-LRR resistance protein; Os04g0621500, encoding for a disease resistance domain containing protein; 3 genes (Os04g0616300, Os04g0616400 and Os04g0616500), representing SHR5-receptor-like kinases, belonging to the VIII-2 subclass of LRR receptor kinases, induced by fungal and bacterial infection in sugarcane and predicted to be involved in plant defense response (Vinagre et al. 2006); Os04g0620000, an ABC transporter, member of a class of transporters implicated in detoxification after fungal infection in wheat (Krattinger et al. 2009; Sucher et al. 2016); three genes (Os04g0624400, Os04g0624450 and Os04g0624500) encoding for polyphenol oxidases, representing a class of enzymes whose over-expression reduced leaf blast severity in rice (Ng et al. 2016); Os04g0615900, a FAR1 domain containing protein, representing a light signaling factor which regulates plant immunity by modulating chlorophyll biosynthesis (Wang et al. 2016); Os04g0616100, corresponding to a tetratricopeptide repeat domain containing protein, and Os04g0618050 encoding for a pentatricopeptide repeat domain containing protein. Both these two domains have been demonstrated to be present in proteins implicated in plant resistance (Spoel and Dong 2012; Sekhwal et al. 2015).

Additional candidate genes for the *bakanae* resistance loci were searched among the Differentially Expressed Genes (DEGs) identified in a previous RNA-Seq comparative transcriptomic analysis, of resistant and susceptible rice cvs. (Selenio and Dorella, respectively), in response to *F. fujikuroi* (Matic et al. 2016). DEGs were selected according to the four criteria listed in the Materials and methods and only those showing a genomic position within or near the two major *bakanae* resistance loci defined above were considered. A single locus (Os01g0112600), encoding for a protein of unknown function and induced by infection in the resistant cv. Selenio (log_2_Fold Change (log_2_FC) values = 2.17), was discovered for *qBK1_628091*, while 18 candidate loci were identified for *qBK4_31750955* (Additional file 8: Table S3). Among them, the Aldo/keto reductase encoding *locus* Os04g0594400 was less expressed in Selenio, with respect to Dorella, in the presence of the fungus at 1 wpg (log_2_FC = −15.21). However, it was repressed by infection in Dorella at 3 wpg (log_2_FC = −2.03), while induction by infection in Selenio at 3wpg was detected (log_2_FC = 1.3), thus leading to a higher level of transcription in the resistant genotype during infection at 3 wpg (log_2_FC = 2.72). Plant aldo-keto reductases are enzymes involved in the response to stresses, including abiotic and biotic challenges (Sengupta et al. 2015). Os04g0598900 encodes for a protein similar to wall-associated kinases and was more expressed in Selenio vs. Dorella in both conditions at 1 wpg (log_2_FCs = 1.01 and 14.275, in mock and infected samples respectively) and during infection at 3 wpg (log_2_FC = 3.11). Moreover, at 3 wpg the gene was repressed by infection in Dorella (log_2_FC = −2.55) and induced by infection in Selenio (log_2_FC = 1.14). Os04g0616400 encoded for a protein similar to a receptor-like serine/threonine kinase and was more expressed in Selenio, with respect to Dorella, in both treatments and time-points of germination (log_2_FCs ranging from 5.37 to 66). Finally, Os04g0652400 represented a protein similar to a sulphate transporter and was more expressed in infected Selenio vs. infected Dorella at both time-points of growth (log_2_FCs = 16.98 and 1.265, respectively at 1 and 3 wpg).

## Discussion

In this work, a panel of rice temperate and tropical *japonica* accessions from different origins, mainly from Italy (67), USA (28), Portugal (11) and Spain (10) was assembled in order to identify new loci associated with resistance to the rice *bakanae* disease through a GWA mapping approach. The analysis of LD decay for each chromosome in this panel showed an average value of 1992 Kb, which is considerably higher than those commonly reported in the literature of about 150–180 kb for *japonica* backgrounds (Mather et al. 2007, Huang et al. 2011, Courtois et al. 2013). However, higher LD values ranging from 600 kb up to 2 Mb were also observed in a number of cases for *japonica* and *indica* rice (Xu et al. 2011, Kumar et al. 2015); moreover, a germplasm collection of more related temperate *japonica* rice accessions recorded values of LD decay of 1250 kb (Biscarini et al. 2016). Since the panel used in this study represents a sub-group of the panel used in Biscarini et al. (2016), it is conceivable that the higher values of LD observed here are most likely due to a lower level of diversity among the varieties included in the present collection, suggesting that few historical recombination events occurred in this population. Moreover, SNP density applied in our study can contribute to the higher LD value estimated; in the present work a total of 31,752 SNPs were used to estimate LD (and for GWAS analysis) while Courtois et al. (2013) used 16,664 markers (both SNPs and DArTs) and Mather et al. (2007) used only 522 markers. LD estimates tend to be higher with denser SNP panels (Khatkar et al. 2008, O’Brien et al. 2014), and LD patterns tend to emerge clearly only at higher SNP densities (Bacciu et al. 2012). The resulting higher LD detected may eliminate true positives if in one region in LD more than one significant association is present, however considering both, the extent of LD decay observed and the expected average marker density (calculated as 0.09 SNP/Kbp in the whole population), we were confident that this panel represented an excellent resource for investigating *bakanae* resistance in *japonica* rice.

Screening of the GWAS panel allowed the identification of accessions with a low disease index (I*); even considering that the disease incidence among temperate and tropical *japonica* accessions was not statistically different, ten of the 12 more resistant accessions (i.e. those showing I* values <27) were identified within the tropical *japonica* background, raising the possibility that higher frequency of effective *bakanae* resistance loci is present in tropical with respect to temperate *japonica*. However, since no screenings for *bakanae* resistance involving relevant numbers of accessions belonging to the different rice groups (temperate and tropical *japonica*, *indica*, *aromatic*, *aus*) have been carried out so far, these conclusions cannot be adequately supported. The GWAS analysis was therefore carried out using a restricted number of related sub-populations (temperate and tropical *japonica*). As previously observed, this approach from one side increases the possibility to detect associations for alleles that are segregating only in one or two populations while are fixed in others, but from the other side the resulting higher LD may eliminate true positives (Famoso et al. 2011; Zhao et al. 2011). However, the high frequency of the resistant phenotypes in the tropical *japonica* sub-population detected in this work, leveraged power to detect alleles that were segregating within this sub-population.

To our knowledge, the present work represents the first report on the utilization of a GWAS approach for the identification of resistant loci effective against the *bakanae* disease of rice. Two genomic regions were associated to *bakanae* resistance and delimited to about 0.41 Mb on chromosome 1 (from position 628,091 to 1,040,823) and 0.59 Mb on chromosome 4 (from position 31,162,467 bp to 31,757,436 bp), and were named as *qBK1_628091* and *qBK4_31750955*, respectively. Different *bakanae* resistance QTLs have been previously located on rice chromosome 1 (Fiyaz et al. 2016; Hur et al. 2015; Yang et al. 2006). Of these, *qBK1.2* and *qBK1.3* (Fiyaz et al. 2016) and *qB1* (Yang et al. 2006) were located on the short arm of chromosome 1. The comparison between our resistance-associated region on chromosome 1 and *qBK1.2*, identified in a 0.26 Mb region between RM10153 and RM5336 (from position 3,105,042 to 3,367,533; Fiyaz et al. 2016), demonstrated that *qBK1_628091* was located apart from *qBK1.2*. Similarly, the region associated to *qBK1.3*, ranging from RM10271 and RM35 (from position 4,657,288 to 8,411,302 bp; Fiyaz et al. 2016), and *qB1*, spanning from RM7180 to RM486 (from position 34,105,454 and 34,956,597 bp; Yang et al. 2006) resulted different from *qBK1_628091*. Moreover, previous studies (Ma et al. 2008) indicated that rice varieties with the *sd1* gene, a semi-dwarf gene resulting in defective 20-oxidase GA biosynthetic enzyme and localized from 38,382,382 to 38,385,504, are susceptible to *bakanae* disease. The detected position for *qBK1_628091* indicates that this resistance *locus* is not related to the *sd1* allele. All these observations therefore indicate that the QTL we have detected on chromosome 1 represents a novel unknown *locus* involved in *bakanae* resistance. Moreover, no *bakanae*-resistance loci have been previously mapped on chromosome 4, suggesting that, also in this case, *qBK4_31750955* represents a new genomic region associated to *F. fujikuroi* resistance.

Alternative alleles for SNPs representing peak markers for the two resistance loci were identified, where the “A” and “C” alleles for *qBK1_628091* and *qBK4_31750955* respectively, were associated to a lower *bakanae* disease incidence. Noteworthy, when the 12 most resistant accessions (with I* value <27) were analyzed, 11 of them carried the combination of the “A” and “C” alleles (for Adair, the C allele at *qBK4_31750955* has been imputed from neighbor markers, data not shown), suggesting that pyramiding of the two loci should provide effective levels of resistance. Within these accessions, the tropical *japonica* sub-group was predominant (10 accessions out of 12); this result, together with the observation that tropical *japonica* have a lower level of average disease incidence, may indicate that higher breeding pressure for *bakanae* resistance was applied in the tropical *japonica* than on the temperate sub-group. This observation is further supported by the higher frequency of the resistant allele (“A”) at the *qBK1_628091* peak marker observed in tropical *japonica* (41.5%) compared to temperate *japonica* (5.1%). Sequences corresponding to the peak markers for *qBK1_628091* and *qBK4_31750955* are here provided (Additional file 6: Figure S5). These sequences can be used to develop SNP-based high-throughput markers to be used in marker-assisted selection for pyramiding the two resistant loci in *bakanae* susceptible lines. Moreover, effective resistance loci were also identified in several different commodity classes, including round (Greppi), long A (Maioral and Bengal) and long B (Arsenal, Adair, King), an aspect that should facilitate the introgression of *bakanae* resistance into rice lines addressing different market requirements.

Several annotated genes encoding functions compatible with resistance were identified for both resistance loci genetic intervals. These included receptor-like kinases, such as LRK10 and LRK14, known to participate to wheat leaf rust resistance (Feuillet et al. 1998) for the *qBK1_628091* region, while for *qBK4_31750955,* a NB-LRR gene, receptor kinases and an ABC transporter were identified. A second approach for identification of candidates was based on the integration of mapping position and RNA-Seq data previously obtained in a comparative transcriptome analysis of resistant and susceptible rice cvs., Selenio and Dorella respectively, in response to *F. fujikuroi* (Matic et al. 2016). DEGs located within or near the two QTLs regions were analyzed according to the criteria indicated in Methods. The analysis did not lead to identification of candidates for *qBK1_628091*, as only one locus encoding for a protein of unknown function fitting the criteria was located on this QTL region. For the *qBK4_*31750955, a SHR5-receptor kinases was listed among the candidate genes using the combined DEGs and map position approaches. Finally, also a gene encoding for a sulphate transporter, transcribed at higher rates in Selenio during infection (Matic et al. 2016), was located in the *qBK4_31750955* region. It is well known that sulphur increases resistance in different plant-fungal pathogen interactions, inducing the production of a number of sulphur compounds implicated in defense responses like glucosinolates, phytoalexins, H_2_S, cysteine and glutathione (Walters and Bingham 2007). Thus, another possible *qBK4_31750955* function might be related to S uptake and related production of S-resistance compounds.

Overall, the *in silico* search for candidate genes, in the two QTL regions (qBK1_628091 and qBK4_31750955) identified in this work, highlighted several genes with functions associated to disease resistance that could represent candidates for *bakanae* resistance. It should however be considered that these genes were identified on the Nipponbare genome and that, currently, the reaction of this rice cv. to *bakanae* infection is not known. Additional investigations involving targeted resequencing of the two QTL regions in resistant and susceptible accessions here identified and the comparison of these regions with the available Nipponbare sequence are therefore required. To address the final identification of the genes responsible for *bakanae* resistance we are developing high-resolution mapping populations for *qBK1_628091* and *qBK4_31750955* through crossing accessions bearing only one of the two loci with highly susceptible accessions. These materials will allow a fine mapping of the two loci and a more detailed and precise assessment of the candidate genes here reported until the identification of the genes underlying the QTL involved in resistance.

## Conclusions

Screening of a *japonica* rice germplasm collection carried out with a virulent *F. fujikuroj* isolate allowed the identification of accessions bearing relevant levels of resistance. The subsequent GWAS approach under stringent conditions identified two previously un-identified *bakanae* resistance loci on the short arm of chromosome 1 and on the long arm of chromosome 4. Since high levels of phenotypic resistance to *bakanae* was associated to the cumulated presence of the peak markers resistance alleles at the two loci, it is expected that they can have an additive effect that could be exploited also in resistance breeding. Candidate genes with a putative role in *bakanae* resistance were identified in the two genomic regions highlighting several gene functions that could be involved in resistance opening the way for the functional characterization of the resistance loci.

## Methods

### Plant materials and genotyping

The accession panel used in this study included 138 *O. sativa* varieties from the Rice Germplasm Collection maintained at the CREA-Rice Research Unit (Vercelli, Italy). The sampled collection included 41 tropical *japonica* and 97 temperate *japonica* accessions. Most of these were temperate rice developed in Italy (67 accessions), selected from larger collections with the aim of including the broadest range of genetic and phenotypic variation (Faivre-Rampant et al. 2011; Biscarini et al. 2016). The remaining 71 genotypes were developed elsewhere but they are considered adapted to Italian agro-climatic conditions. The complete list of accessions used in this study, with information on taxonomic group and geographic origin, is reported in Table 1.

All the accessions were subjected to genotyping-by-sequencing (GBS) as described by Biscarini et al. (2016). The analysis yielded a set of 166,418 SNP markers, which were filtered for call rate (1 - percentage of missing data) and minor allele frequency (MAF) with the PLINK software (http://zzz.bwh.harvard.edu/plink/; Purcell et al. 2007). Different filtering thresholds were chosen depending on the analysis performed.

### Phenotyping for *bakanae* resistance

The collection of 138 rice genotypes was evaluated for *bakanae* resistance after seed inoculation. For the inoculum production, the ER 2103 *F. fujikuroi* isolate from the CREA-PAV collection was used. This isolate was previously tested for its virulence by seed inoculation of the susceptible rice cv. Galileo, by the same method described below for phenotyping of the whole rice collection. The fungus was grown for 3 days on 20% V8 juice liquid medium (Miller 1955) with shaking (120 rpm) at 23 °C, microconidia were harvested and the concentration was adjusted to 10^6^ spores ml^−1^. Rice seeds were surface sterilized in 70% ethanol for 1 min. With shacking, then in 1.5% sodium hypochlorite for 30 min and subsequently rinsed 5 times in sterile water. Surface sterilized seeds were inoculated by dipping the seeds in the inoculum for 30 min, immediately before sowing in pots containing soil; therefore, temperatures applied for inoculums were the same as those applied to growth the rice plantlets, below indicated. A complete randomized block design with three replicates and 25 seeds for each replicate was used. Plants were kept in the greenhouse at 20–25 °C with a 12 h photoperiod. After 30 days, seedlings were evaluated for symptoms by using the 0–4 disease scale of Mohd Zainudin et al. (2008) with the following modifications: 0 = no symptoms; 1 = normal growth but leaves beginning to show yellowish-green, small necrosis localized at the crown level; 2 = abnormal growth, elongated, thin and yellowish-green leaves; seedlings also shorter or taller than normal, necrosis on main root and crown; 3 = abnormal growth, elongated, chlorotic, thin and brownish leaves; seedlings also shorter or taller than normal, reduced root system with necrosis on secondary roots and on basal stem; 4 = dead plants. Based on scoring values of each plant, gravity index was assessed by using McKinney index (I; McKinney 1923) calculated as:

$$ I=\frac{\sum \left( f\ast v\right)}{N\ast X}\ast 100 $$

where: f = value of the scoring class (0, 1, 2, 3, 4), v = number of plants of each class, N = total of observed plants, X = highest value of the evaluation scale. Data were arcsin transformed (disease index, I*) prior to analysis of variance (ANOVA). Two-factor ANOVA was carried out with the software GenStat (Payne et al. 2009) to evaluate differences for *bakanae* resistance between genotypes. Broad sense heritability (H^2^) was estimated from the variance components obtained by fitting both replications and genotypes as random terms as H^2^ = σ^2^_g_/(σ^2^_g_ + σ^2^_e_), where σ^2^_g_ is the genotypic variance component and σ^2^_e_ is the residual variance component.

### Analysis of population structure and genetic diversity

The panel of rice varieties was screened for the presence of sub-populations using a model-based approach integrated by neighbor joining phylogenetic and Principal Coordinate (PCoA) analyses. For this purpose we used a subset of 10,000 SNP markers randomly selected from the whole dataset by applying the following thresholds: call rate 95%, MAF 5%.

The model-based analysis was performed using Structure v2.3.4 (Pritchard et al. 2000). The data were analyzed as haploids (a correct approach for a highly autogamous species such as rice; Nordborg et al. 2005). The following parameters were used: presence of admixture admitted; allele frequencies among sub-populations correlated; 20,000 burn-in cycles followed by 10,000 Monte Carlo – Markov Chain (MCMC) iterations; a number of sub-populations (K) ranging between 1 and 8; 5 runs per K value. The results of the Structure analysis were analyzed according to Evanno et al. (2005) with the Structure Harvester program (Earl and vonHoldt 2012) to identify the most probable number of clusters in the population. The changing of the population clustering (number of sub-populations, number of admixed accessions) was also evaluated at increasing values of K, as proposed by Courtois et al. (2012). Once defined this parameter, one single run of the Structure analysis was repeated at the most probable K value to maximize the accuracy in determining the membership of each accession. The same parameters as above were used, except for the number of burn-in and MCMC iterations (150,000 and 100,000 respectively). Accessions with membership coefficients ≥0.7 were assigned to a specific sub-population, whereas the remaining genotypes were identified as admixed.

A neighbor-joining tree was built with the MEGA v7 software (Kumar et al. 2016), based on the Jukes-Cantor model which is appropriate for sequence data when the rate of nucleotide substitution is expected to be equal for all pairs of the four nucleotides. Bootstrap values (300×) were computed and added to the tree branches when higher than 70% (Hillis and Bull 1993). The resulting tree was imported in iTOL (http://itol.embl.de/; Letunic and Bork 2016) and implemented with the provenience information (Faivre-Rampant et al. 2011; Biscarini et al. 2016; Table 1) and with the results of the above Structure analysis.

Finally, a PCoA was performed with the PAST v3.11 software (Hammer et al. 2001) with the Jukes-Cantor algorithm; sub-population attributions derived from Structure analysis and taxonomic groups defined in the literature (Biscarini et al. 2016; Courtois et al. 2012) were projected onto the final output.

For the genetic diversity analyses, the number of polymorphic loci, the expected heterozygosity (He; Nei 1978) and the number of transitions and transvertions were computed using the Arlequin v3.5 software (Excoffier and Lischer 2010). The whole sample and the following partitions of the accessions were considered for the analyses: temperate *japonica*, tropical *japonica* and, within the tropical *japonica,* sampling was done according to the provenience (Europe and USA). The genetic diversity statistics described above were also computed for the genetic groups highlighted by the Structure analysis. The divergence among the populations defined a priori according to the subspecies, within tropical *japonica* and among groups identified by Structure, was estimated as FST (Weir and Cockerham 1984). The significance of the estimates was obtained through permutation tests, using 1000 permutations. The Arlequin v3.5 software (Excoffier and Lischer 2010) was used.

### Analysis of linkage disequilibrium and association mapping

The expected resolution of the association mapping panel was evaluated by calculating the linkage disequilibrium (LD) as the correlation (R^2^) between *loci* on each chromosome, after filtering the SNP markers with the following threshold values: call rate > 95%; MAF > 5%.

The R^2^ computation was performed with the package LDcorSV v1.3.1 (https://cran.r-project.org/web/packages/LDcorSV/index.html) implemented in R; the values were therefore plotted against physical distance and fitted to a second degree LOESS curve (Cleveland 1979, Marroni et al. 2011) using the R language. A critical value of 0.2 was set as R^2^ between unlinked *loci*. The value of physical distance corresponding to a LOESS curve value of 0.2 was assumed as an estimate of the LD extent in each chromosome.

Genome-wide association between markers with call rate > 95% and minor allele frequency > 10% and the phenotypic data was performed by fitting a Mixed Linear Model (MLM) in Tassel v5.0 (Bradbury et al. 2007), that includes a kinship matrix as random term to account for genetic relatedness due to population structure. MLM was run with the optimal compression and genetic and residual variances were estimated for each SNP marker. False Discovery Rate (FDR) was calculated with the R package q-value (http://qvalue.princeton.edu) in order to detect significant SNP associations. Finally, the R package qqman (https://cran.r-project.org/package=qqman) was used to draw Manhattan plots.

### Search for candidate genes

The genomic regions associated to *bakanae* resistance have been selected on the base of FDR value (i.e. regions defined by significantly associated markers) and used as starting point to explore the genomic context of the *Oryza sativa* reference sequence (Os-Nipponbare-Reference-IRGSP-1.0, http://rapdb.dna.affrc.go.jp/download/irgsp1.html). All annotated genes included in the selected genomic windows have been scanned to identify candidate genes.

Additional candidate resistance genes were identified among the Differentially Expressed Genes (DEGs), located on the selected genomic regions, from a RNA-Seq comparative transcriptome analysis of resistant and susceptible rice cvs. Selenio and Dorella respectively, in response to *F. fujikuroi* at one and 3 weeks post-germination (Matic et al. 2016). DEGs were selected according to the following criteria: a) induction by infection in the resistant genotype only and higher expression in the resistant cv. with respect to the susceptible during infection; b) induction by infection in both genotypes and higher expression in the resistant cv., with respect to the susceptible during infection; c) higher expression in the resistant genotype with respect to the susceptible in mock conditions, but not infection responsiveness; d) induction by infection in the resistance genotype and repression by infection in the susceptible cv. and higher expression in resistant vs. susceptible comparisons.

## Additional files

Additional file 1: Figure S1. Structure Harvester analysis. Four different parameters are reported to evaluate the most probable number of subpopulations in the panel used for GWAS. Ln(k) = mean of the likelihood distribution LnP(D) over 5 runs for each K value from 1 to 7; Ln’(K) = rate of change of the likelihood function with respect to K; |Ln”(K)| = second order rate of change of Ln(K) with respect to K; Delta(K) = mean(|Ln”(K)|)/DevSt(L(K)). (TIFF 205 kb)

Additional file 2: Figure S2. Principal Coordinate Analysis of the Rice Germplasm Collection; each point shape represents a different cluster obtained in the STRUCTURE analysis at K = 2 (top panel) or a different taxonomic group defined in the literature (bottom panel) (see the text for details). (TIFF 355 kb)

Additional file 3: Table S1. Genetic diversity (A), Genetic divergence (FST) (B) and marker density (C) estimates computed for the whole rice panel and for the major subdivisions of the panel. (PDF 49 kb)

Additional file 4: Figure S3. Details of the two QTL (on chromosomes Os1 and Os4) associated to *bakanae* disease resistance in the Rice Germplasm Collection. The horizontal line represents the 0.01 FDR threshold and defines the genomic regions where candidate genes were searched for. (TIFF 401 kb)

Additional file 5: Figure S4. Box-plots showing the phenotypic distributions for the alternative alleles at the most significant SNPs detected in this work. (TIFF 132 kb)

Additional file 6: Figure S5. Sequences encompassing the peak SNP markers detected for *bakanae* disease resistance on chromosomes 1 and 4. The alleles associated with the resistant phenotype are in bold. (PDF 372 kb)

Additional file 7: Table S2. List of candidate genes, functionally annotated, selected on the base of the FDR value. (XLSX 14 kb)

Additional file 8: Table S3. List of candidate genes selected from Differentially Expressed Genes (DEGs). (XLSX 13 kb)
