# Supplementary figures and images for: Identification of bakanae disease resistance loci in japonica rice through genome wide association study
Source: Rice (N Y). 2017 Jun 8;10:29. doi: 10.1186/s12284-017-0168-z (PMC5465229; doi:10.1186/s12284-017-0168-z)

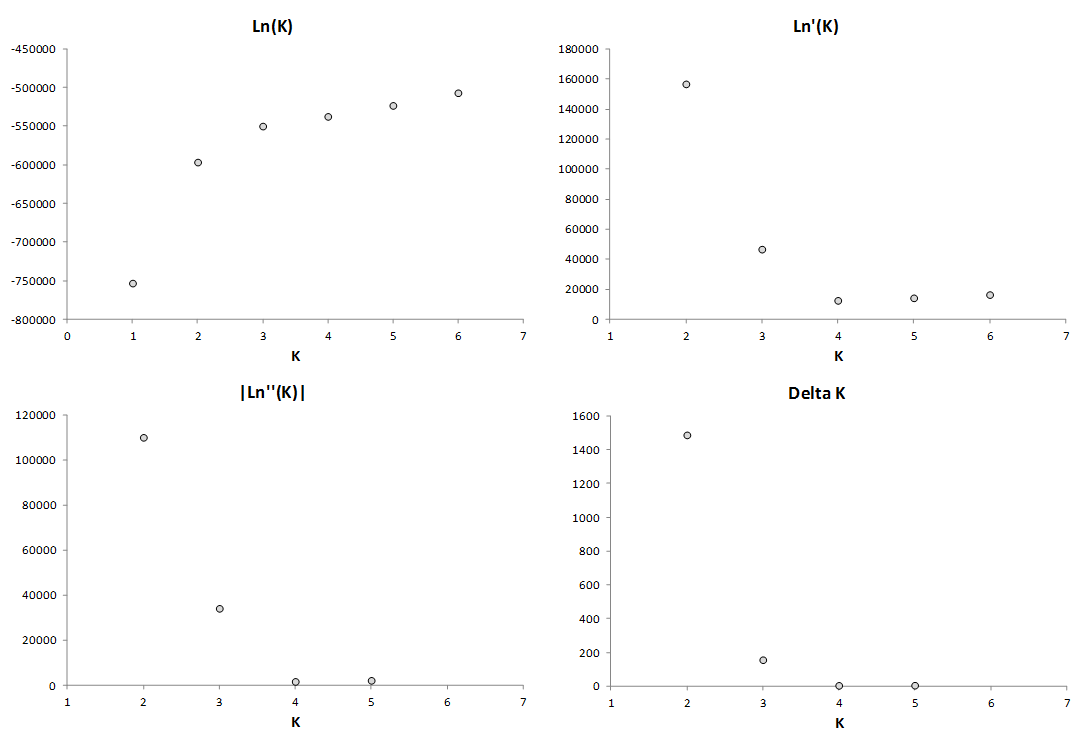

Supplement: Supplementary file 1 — Structure Harvester analysis. Four different parameters are reported to evaluate the most probable number of subpopulations in the panel used for GWAS. Ln(k) = mean of the likelihood distribution LnP(D) over 5 runs for each K value from 1 to 7; Ln’(K) = rate of change of the likelihood function with respect to K; |Ln”(K)| = second order rate of change of Ln(K) with respect to K; Delta(K) = mean(|Ln”(K)|)/DevSt(L(K)). (TIFF 205 kb) [file 12284_2017_168_MOESM1_ESM.tif]

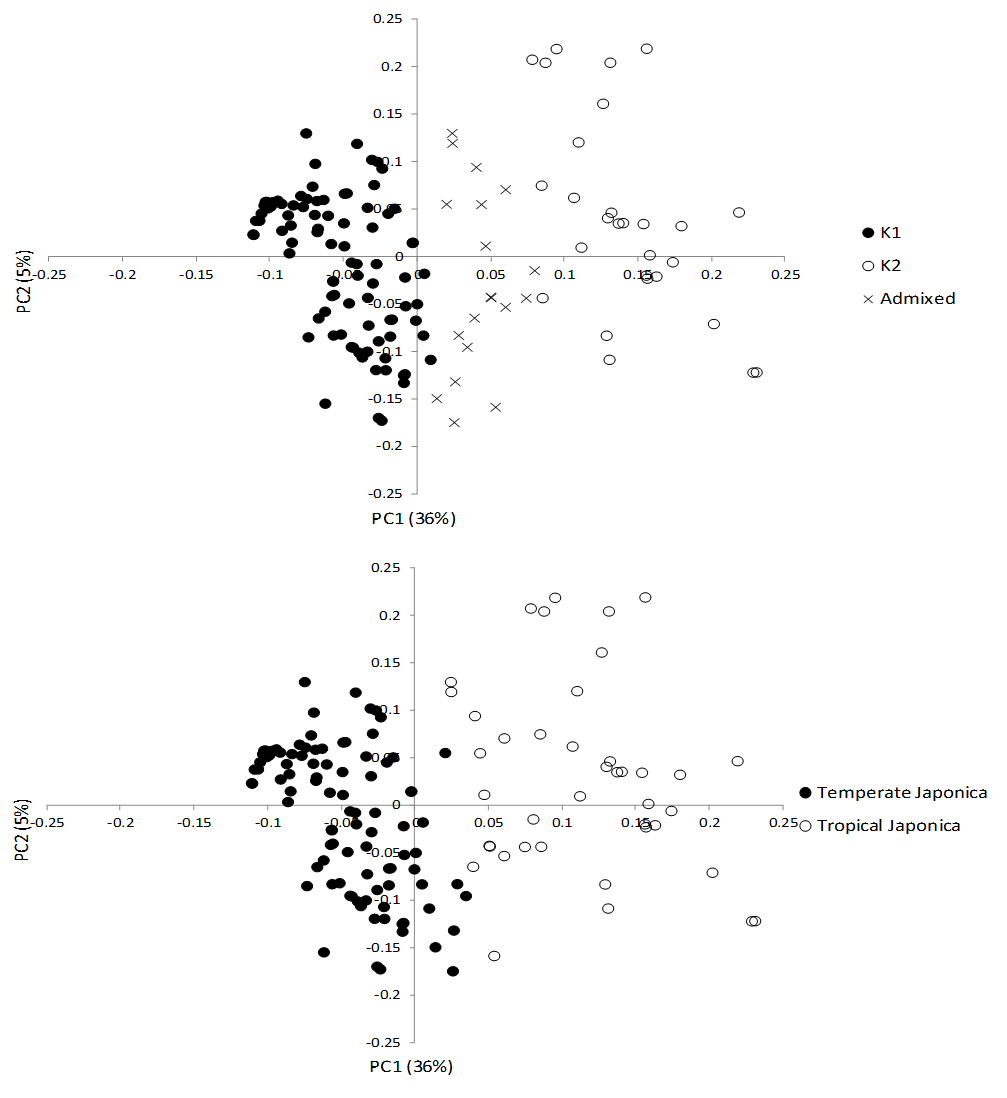

Supplement: Supplementary file 2 — Principal Coordinate Analysis of the Rice Germplasm Collection; each point shape represents a different cluster obtained in the STRUCTURE analysis at K = 2 (top panel) or a different taxonomic group defined in the literature (bottom panel) (see the text for details). (TIFF 355 kb) [file 12284_2017_168_MOESM2_ESM.tif]

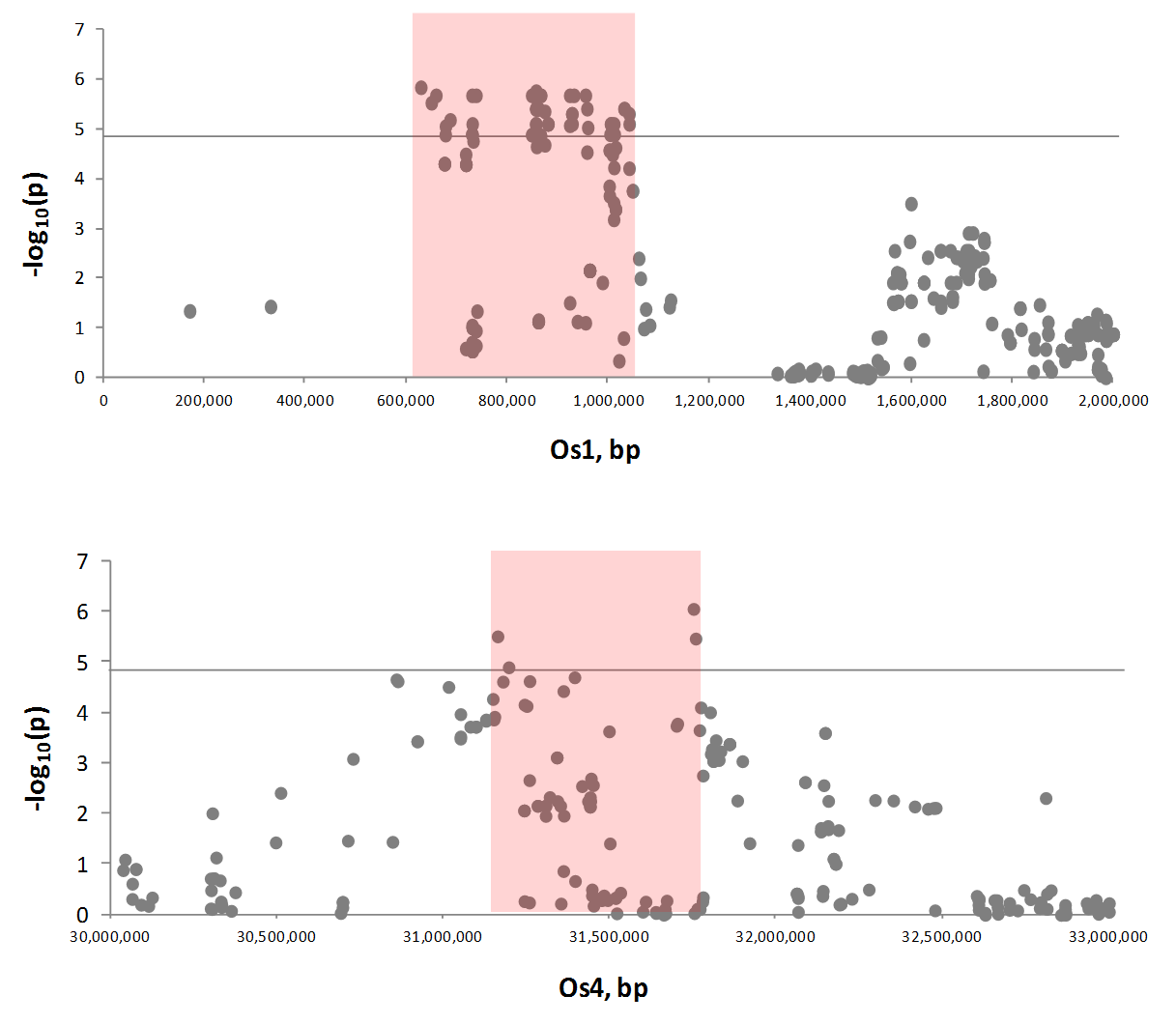

Supplement: Supplementary file 4 — Details of the two QTL (on chromosomes Os1 and Os4) associated to bakanae disease resistance in the Rice Germplasm Collection. The horizontal line represents the 0.01 FDR threshold and defines the genomic regions where candidate genes were searched for. (TIFF 401 kb) [file 12284_2017_168_MOESM4_ESM.tif]

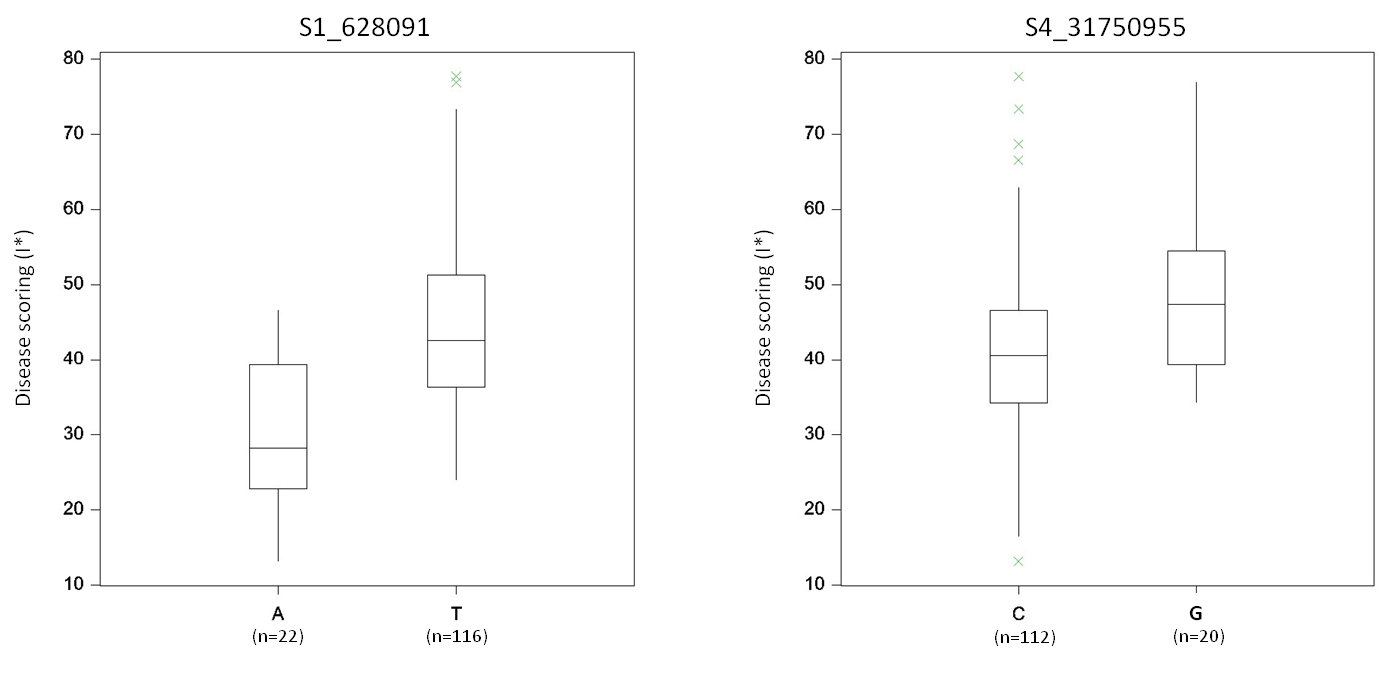

Supplement: Supplementary file 5 — Box-plots showing the phenotypic distributions for the alternative alleles at the most significant SNPs detected in this work. (TIFF 132 kb) [file 12284_2017_168_MOESM5_ESM.tif]
