# Supplementary material for: Identification of bakanae disease resistance loci in japonica rice through genome wide association study
Source: Rice (N Y). 2017 Jun 8;10:29. doi: 10.1186/s12284-017-0168-z (PMC5465229; doi:10.1186/s12284-017-0168-z)
Supplement: Supplementary file 3 — Genetic diversity (A), Genetic divergence (FST) (B) and marker density (C) estimates computed for the whole rice panel and for the major subdivisions of the panel. (PDF 49 kb) [file 12284_2017_168_MOESM3_ESM.pdf]

**Additional File 3: Table S1.** Genetic diversity (**A**), Genetic divergence (FST) (**B**) and marker density (**C**) estimates computed for the whole rice panel and for the major subdivisions of the panel

**A)**

| | All | Temperate Japonica | Tropical Japonica | Tropical Japonica EUROPE | Tropical Japonica USA | K1 | K2 |
|---|---|---|---|---|---|---|---|
| **No of accession** | 138 | 97 | 41 | 18 | 23 | 91 | 28 |
| **No. of usable loci (call rate > 95%)** | 9856 | 9824 | 8855 | 7528 | 8529 | 9869 | 8225 |
| **No. of polymorphic sites** | 9856 | 9468 | 8484 | 6756 | 6894 | 8663 | 6950 |
| **Nucleotide diversity** | 0.31± 0.15 | 0.23± 0.11 | 0.27± 0.13 | 0.32± 0.16 | 0.22± 0.11 | 0.22± 0.10 | 0.23± 0.11 |
| **No. of transitions** | 6593 | 6341 | 5665 | 4482 | 4600 | 5805 | 4643 |
| **No. of transversions** | 3263 | 3127 | 2819 | 2274 | 2294 | 2858 | 2307 |

**B)**

1)

| Fst | Temperate Japonica | Tropical Japonica |
|---|---|---|
| **Temperate Japonica** | 0 | |
| **Tropical Japonica** | 0.38 | 0 |

2)

| Fst | Temperate Japonica | Tropical Japonica EUROPE | Tropical Japonica USA |
|---|---|---|---|
| **Temperate Japonica** | 0 | | |
| **Tropical Japonica EUROPE** | 0.28 | 0 | |
| **Tropical Japonica USA** | 0.47 | 0.1 | 0 |

3)

| Fst | Admixed | K1 | K2 |
|---|---|---|---|
| **Admixed** | 0 | | |
| **K1** | 0.22 | 0 | |
| **K2** | 0.19 | 0.49 | 0 |

**C)**

| | All | Temperate Japonica | Tropical Japonica | Tropical Japonica EUROPE | Tropical Japonica USA |
|---|---|---|---|---|---|
| **No of accession** | 138 | 97 | 41 | 18 | 23 |
| **No. of loci (call rate > 95%, MAF>10%)** | 31752 | 19953 | 20875 | 20821 | 13393 |
| **N. marker/Mb** | 85.1 | 53.5 | 56.0 | 55.8 | 35.9 |
| **N. marker/Kb** | 0.09 | 0.05 | 0.06 | 0.06 | 0.04 |
