# Supplementary material for: Identification of bakanae disease resistance loci in japonica rice through genome wide association study
Source: Rice (N Y). 2017 Jun 8;10:29. doi: 10.1186/s12284-017-0168-z (PMC5465229; doi:10.1186/s12284-017-0168-z)
Supplement: Supplementary file 6 — Sequences encompassing the peak SNP markers detected for bakanae disease resistance on chromosomes 1 and 4. The alleles associated with the resistant phenotype are in bold. (PDF 372 kb) [file 12284_2017_168_MOESM6_ESM.pdf]

Additional File 6: Figure S5. Sequences encompassing the peak SNP markers detected for *bakanae* disease resistance on chromosomes 1 and 4. The alleles associated with the resistant phenotype are in bold.

qBK1_628091bp (627900-629199)
GAGGACATCCAAAATGACGATGGAGTCGCACTCGATCGACCGGTCGGTGGTTGCCGCGTTGCTGTCAACG
ATTGAACCGTGTTTTAATTCCATGCCTGCTTGTATTATTAATTTATTTTATTAGCTTTTAAAGAAAATTATCAG
GAGAGCTGCTAAGATGGGTAGAGTTCAGAAAGAACAGTTGCTGATGC[T/**A**]TCCATGATACCTAAACCAGT
CCAGTCTAGTCGTTCTACTTTACTGGCAGCTAGTACCAATTTGACTTGTTCTTGTACTAGTACTACTGCTCCTT
TGGACTTGGAAGAG

qBK2_31750955bp (31750800-31750199)
GTGTTCTTCGCGCACCACGGCAACGTCGACCGCATGTGGCACATCCGCCGCGGCCTCCTCTTCCCCGGCGAC
ACCGACTTCACCGACCCCGACTGGCTCGACGCCAGCTTCTTCTTCTACGACGAGGAGGCCCGCCTCGTCCGC
GTCCGCGTCCG[G/**C**]GACACCCTCGACCCGTCGGCGCTGCGCTTCACGTACCAGGACGTGGGTCTCCCGTG
GCTGAACGCCAAGCCGTCCACGGGAGCAGCCAGCACGCCGGCGCCCGCGGCCGGCGCGTTCCCGGCGACC
CTGGACAAGACCGTGCGG
